# Autonomy-Supportive Classroom Climate: Its Multilevel Structure and Associations with Students’ Mathematics Achievement

**DOI:** 10.3390/bs16081268

**Published:** 2026-07-23

**Authors:** Sara Manganelli, Giulia Raimondi, Fabio Alivernini, James Dawe, Elisa Cavicchiolo

**Affiliations:** 1 Department of Developmental and Social Psychology, Sapienza University of Rome, Via dei Marsi 78, 00185 Rome, Italy; sara.manganelli@uniroma1.it (S.M.); fabio.alivernini@uniroma1.it (F.A.); 2 BeSSA Department, Wellbeing, Health and Environmental Sustainability, Sapienza University of Rome, Via delle Fontanelle, 02100 Rieti, Italy; 3 Department of Humanities and Social Sciences, Universitas Mercatorum Telematic University, Piazza Mattei 10, 00186 Rome, Italy; james.dawe@unimercatorum.it; 4 Department of Systems Medicine, Tor Vergata University of Rome, Via Montpellier 1, 00133 Rome, Italy; elisa.cavicchiolo@uniroma2.it

**Keywords:** autonomy support, Self-Determination Theory, motivational climate theory, multilevel, doubly latent multilevel SEM, math achievement

## Abstract

Background: Autonomy-supportive climate has consistently been associated with positive student outcomes. Although Self-Determination Theory and Motivational Climate Theory conceptualize it as a multilevel construct, research has mainly examined autonomy support from an individual student perspective. The present study investigated autonomy-supportive climate as both a classroom-level construct and an individual microclimate component, examining its associations with mathematics achievement. Methods: Data came from a representative sample of 26,564 Italian 10th-grade students. Doubly Latent Multilevel Structural Equation Modeling (DL-ML-SEM) was used to examine the multilevel measurement structure of autonomy-supportive climate and its associations with mathematics achievement at the student and classroom levels, controlling for students’ sex, immigrant background, and school type. Results: Multilevel CFA supported the factorial validity and cross-level invariance of the autonomy-supportive climate measure. ICC analyses indicated substantial between-class variability and high reliability of aggregated classroom perceptions. DL-ML-SEM showed that autonomy-supportive climate was positively associated with mathematics achievement at the classroom level, but not at the individual student level. Conclusions: These findings highlight the relevance of examining autonomy support as a classroom-level phenomenon and provide new evidence on the multilevel nature of autonomy-supportive climate in educational settings.

## 1. Introduction

Self-Determination Theory (SDT; Ryan & Deci, 2017) posits that students show higher-quality engagement, greater well-being, and improved learning outcomes when teachers provide autonomy support by satisfying basic psychological needs and fostering autonomous motivation. According to SDT, an autonomy-supportive learning climate emerges when teachers adopt a student-centered orientation and an understanding interpersonal style (Reeve & Cheon, 2021). Motivational climate theory (Robinson, 2023) further suggests that students’ perceptions of autonomy support are multilevel, comprising both shared classroom-level beliefs about teachers’ autonomy-supportive practices and individual student specific perceptions that deviates from these shared perceptions. However, existing research has predominantly focused only on the individual level of students and has often failed to adequately account for the measurement model of autonomy supportive climate. As a result, the multilevel nature and effects of autonomy-supportive climate remain largely underexplored, thereby limiting confidence in conclusions about its impact on student outcomes. To address these limitations, the present study, grounded in SDT and Motivational climate theory, investigates the multilevel structure of autonomy-supportive climate in classroom and its influences on students’ math achievement applying Doubly Latent Multilevel Structural Equation Modeling (DL-ML-SEM; Marsh et al., 2012; Morin et al., 2014) on a large representative sample of Italian 10th-grade students. In the remainder of this paper, we first give a brief overview of the research literature that forms the basis of our study. Second, we describe the aims, methods, and results of our empirical study. Finally, we discuss our main results, also examining their implication for future research and practical applications.

### 1.1. Theoretical and Empirical Background

Autonomy lies at the core of SDT framework, as it underpins students’ intrinsic motivation and their willingness to take initiative and engage actively in classroom activities (Ryan & Deci, 2017). It further facilitates the internalization of external regulations, the pursuit of personal growth and goal setting, and overall psychological well-being. Teachers can support students’ autonomy by identifying, nurturing, and developing their internal motivational resources (e.g., interests, preferences, and psychological needs), by adopting a student-centered orientation and an understanding interpersonal style (Ryan & Deci, 2017). More specifically, autonomy-supportive teaching strategies include taking the students’ perspective, supporting their interests and preferences, offering meaningful choices, clarifying learning objectives, using engaging activities and resources, providing explanatory rationales that highlight the personal relevance of tasks, and acknowledging potential difficulties students may encounter (Ahmadi et al., 2023; Reeve & Cheon, 2021).

Numerous studies, including experimental research, have consistently shown that an autonomy-supportive teaching fosters self-determined motivation and a wide range of positive outcomes, including engagement, performance, and well-being (Ryan & Deci, 2017). Recent longitudinal studies have further indicated that autonomy supportive teaching can foster academic motivation and reduce drop out intentions among students from low-income families (Alivernini et al., 2023; Cavicchiolo et al., 2026). With respect to academic achievement, recent longitudinal studies at the individual student level have shown that teachers’ autonomy support is positively associated with students’ mathematics achievement (Fu et al., 2023; Wei et al., 2020; Wei & Zhang, 2025). This association has been explained by the fact that, by satisfying students’ need for autonomy, teachers can enhance students’ intrinsic and autonomous motivation, which in turn fosters greater engagement in learning and improved academic outcomes (Fu et al., 2023). Moreover, the student-focused attitude adopted by autonomy-supportive teachers promotes greater emotional security, higher-quality relationships with students, and a stronger sense of competence among students. All these experiences further support the satisfaction of students’ basic psychological needs, making the learning experience more effective and productive (Reeve & Cheon, 2021).

Motivational Climate Theory (Robinson, 2023) identifies autonomy-supportive climate as one of the central dimensions of classroom motivational climates and microclimates (Johnson et al., 2024). According to this theory, students’ shared perceptions of the autonomy support provided by teachers constitute the classroom autonomy-supportive climate, whereas individual students’ perceptions that diverge from these shared perceptions constitute the autonomy-supportive microclimate. Classroom autonomy-supportive climate refers to a shared mental representation, or classroom culture, in which students recognize common experiences of autonomy-supportive teaching behaviors that characterize and distinguish one classroom from another. From an assessment perspective (Lüdtke et al., 2008; Morin et al., 2014), classroom climate constructs require students to evaluate external characteristics of the classroom environment that are shared by all students, rather than their own personal characteristics or experiences. Nevertheless, individual students may differ in how they perceive autonomy-supportive teaching behaviors, resulting in appraisals that diverge from these shared classroom perceptions (Robinson & Lee, 2023). Such discrepancies have been regarded as a source of measurement unreliability because, within a given classroom, students’ ratings are expected to reflect the same underlying classroom-level construct (Lüdtke et al., 2008; Morin et al., 2014). However, it has also been noted that, once the multilevel structure of classroom climate data is appropriately accounted for statistically using ML-DL-SEM, these unique perceptions may represent meaningful individual differences that contribute to the interpretation of classroom climate and its associations with educational outcomes (Morin et al., 2014). More recently, motivational climate theory has emphasized the importance of these divergent individual perceptions by conceptualizing them as the classroom microclimate (Robinson, 2023). For example (Parrisius et al., 2022), students may differ in their need for autonomy support and therefore perceive the same teacher behaviors as more or less supportive. Likewise, teachers may interact differently with individual students, resulting in partially differing experiences of autonomy support within the same classroom. Consequently, recent reviews of motivational climate research have recommended examining relationships at both the classroom (climate) and individual (microclimate) levels to achieve a more comprehensive understanding of motivational processes and to more accurately capture the complexity of motivational climate (Johnson et al., 2024).

Importantly, both classroom climate and microclimate are conceptualized as subjective psychological phenomena that are distinct from objectively observable classroom practices and teachers’ instructional behaviors. In other words, teachers’ actual instructional practices are defined as separate from students’ perceptions and interpretations of those practices (Robinson & Johnson, 2025). This distinction is supported by literature showing that the extent to which motivational climate and microclimates reflect the motivational supports available in a classroom depends on whether students perceive these supports and appraise them as personally meaningful and relevant (e.g., Vansteenkiste et al., 2020). Moreover, research showed that students’ perceptions play a pivotal role in mediating the relationships between classroom environmental characteristics and educational outcomes (see Robinson, 2023, for a review). From this perspective, students’ subjective perceptions of classroom support may ultimately be more consequential for educational outcomes than the objective characteristics of the support itself. Consistent with this perspective, a recent review of instructional quality (Senden et al., 2021) highlighted that ratings provided by different informants (i.e., students, teachers, or external observers) capture different aspects of the instructional environment. The review further suggests that students’ ratings are particularly valuable because they allow researchers to assess both individual perceptions and shared classroom-level perceptions, provided that the hierarchical structure of the data and the reliability of students’ ratings are appropriately taken into account. Furthermore, because students are repeatedly exposed to instruction over extended periods, across different subjects, teachers, and teaching styles, they are uniquely positioned to evaluate the classroom environment, effectively making them “experts” on their own educational experiences.

The multilevel conceptualization of autonomy-supportive climate captures the complexity of classroom influences on students’ experiences, acknowledging that teachers may direct autonomy-supportive practices toward the class as a whole or vary them across students, and that students within the same classroom may experience the environment differently depending on their readiness, needs, and prior experiences (Robinson, 2023; Robinson & Johnson, 2025). Research has shown that both shared classroom perceptions and individual deviations from those shared perceptions play distinct roles in shaping students’ learning experiences and outcomes (Robinson, 2023).

Nevertheless, most research on the effects of autonomy-supportive teaching on student outcomes (see Johnson et al., 2024, for a systematic review), including the studies examining its association with students’ achievement (e.g., Fu et al., 2023; Wei et al., 2020; Wei & Zhang, 2025), has focused exclusively on the individual student level, without considering the multilevel structure of autonomy-supportive climate. In addition to precluding the examination of classroom climate and microclimate effects, this approach fails to account for the nested structure of the data (i.e., students nested within classrooms), potentially resulting in biased parameter estimates (e.g., Moerbeek, 2004; McNeish, 2023). More recently, some studies have addressed this limitation by using multilevel modeling techniques to investigate the effects of autonomy-supportive climate on a range of outcomes at both the student and classroom levels (e.g., Cheon et al., 2022; Cheon et al., 2023; Jang et al., 2016; Xu et al., 2025). However, none of these studies has examined the effects on students’ achievement. Moreover, to date, no study has preliminarily validated the measurement model of autonomy-supportive climate in a multilevel framework. Indeed, even recent studies that examined it within multilevel contexts either did not report information on its measurement properties (e.g., Jang et al., 2016; Xu et al., 2025) or examined its structure jointly with scales measuring other constructs (e.g., Cheon et al., 2022; Cheon et al., 2023).

### 1.2. The Present Study

Despite the growing literature highlighting the importance of autonomy supportive climate on multiple positive students’ outcomes, several research gaps remain. To address these limitations, the present study draws on a large representative sample of 26,564 Italian 10th-grade students and applies Doubly Latent Multilevel Structural Equation Modeling (DL-ML-SEM; Marsh et al., 2012; Morin et al., 2014) to: (1) empirically examine the multilevel structure of autonomy-supportive climate while estimating between- and within-classroom variability; and (2) investigate the associations between autonomy-supportive climate and students’ math achievement at both the individual and classroom levels while explicitly accounting for measurement error.

With regard to the first aim, examining the multilevel measurement model of autonomy-supportive climate assessed is essential because it enables researchers to determine how the construct functions at both the classroom and individual student levels, thereby allowing for a more accurate estimation of the effects of autonomy supportive climate. In addition, this analysis can provide important information about the extent to which variability in autonomy-supportive climate lies within versus between classrooms, as well as whether students within the same classroom show consensus or heterogeneity in their perceptions of autonomy support.

With regard to the second aim, DL-ML-SEM was employed because it appropriately reflects the theoretical framework adopted in the present study and offers three advantages over alternative analytical techniques (Marsh et al., 2012; Morin et al., 2014). First, it controls for measurement error through the latent aggregation of multiple indicators. Second, it controls for sampling error by using different students’ perceptions as multiple indicators of a latent class-level construct. Third, it enables analysis at the appropriate level by decomposing the variance in students’ responses into student- and class-level components. Therefore, in the present study, DL-ML-SEM allows for a more accurate estimation of the association between autonomy-supportive climate and mathematics achievement, providing for the first time clear distinction between climate and microclimate effects.

The use of a very large and representative sample of classrooms and students constitutes a particular strength of the present study because it makes it possible to employ statistical techniques appropriate for handling the clustered structure of the data and addressing multilevel research questions, while also ensuring substantial participant variability compared with previous studies. Indeed, prior research has shown that studies on classroom climate often rely on samples predominantly composed of female students and/or White, socioeconomically advantaged, and highly educated populations, thereby limiting the generalizability of findings to more diverse student populations (Johnson et al., 2024). To control for potential confounding effects and estimate the unique association between classroom autonomy-supportive climate and mathematics achievement, we included students’ demographic characteristics (sex and immigrant background) and a contextual variable (school type) as covariates, as previous research has shown that these variables are associated with mathematics achievement. Since teaching practices are inherently classroom-level phenomena, the classroom represents the most immediate and theoretically relevant clustering unit for the variables of interest. Moreover, given the high degree of instructional autonomy in the Italian context (OECD, 2025), variation in autonomy-supportive teaching practices is expected to occur primarily between teachers/classrooms rather than between schools. For these reasons, the present study adopts a two-level model with students nested within classrooms.

## 2. Materials and Methods

### 2.1. Participants and Procedures

The data analyzed in this study were drawn from a representative sample of 26,564 Italian 10th-grade students who participated in the National Evaluation of Learning (INVALSI, 2015). Entire classes of tenth-grade students were randomly selected from the population of Italian public upper secondary schools to take part in the survey. The mean age of the students was 15.60 years (SD = 0.76); 49% were male, 6.7% were first-generation immigrants (born abroad to foreign-born parents), and 5.7% were second-generation immigrants (born in Italy to parents both born abroad).

All students in the sampled classes completed standardized assessments developed by INVALSI (2015), which measured math achievement, along with a questionnaire comprising the Learning Climate Questionnaire and demographic variables. The questionnaires were administered in the classroom during the first part of the school day. The assessment adhered to the ethical guidelines of INVALSI (INVALSI, 2015), which reviewed and approved the assessment process. In accordance with INVALSI assessment protocol, each participating school secured informed consent and parental authorization. The dataset used in the present study can be accessed upon request at: https://serviziostatistico.invalsi.it/en/ (accessed on 10 January 2025).

### 2.2. Measures

Autonomy-supportive climate was measured by using four items of Italian version (Alivernini et al., 2019) of the Learning Climate Questionnaire (LCQ; Williams & Deci, 1996; Black & Deci, 2000). The students indicated to what extent their teachers’ behaviors could be described according to four statements on a scale ranging from 1 (strongly disagree) to 4 (strongly agree). Following the instructions provided for the use of this questionnaire to assess the general learning climate (Perceived Autonomy Support: The Climate Questionnaires, 2012), the items asked students to report on the autonomy-supportive behaviors of teachers in their classroom as a whole, rather than on the behavior of a teacher of a specific subject (e.g., mathematics). In addition, consistent with methodological recommendations for the assessment of classroom climate constructs (Morin et al., 2014; Robinson & Johnson, 2025), the items were reworded to position students as observers of the classroom climate rather than as recipients of teachers’ behaviors directed toward themselves.

Due to the limited number of items that could be included in this large-scale national assessment, we selected four of the six items from the LCQ. The selected items were chosen to maximize the content coverage of the construct by representing the two core dimensions of autonomy-supportive teaching identified by Reeve and Cheon (2021): a student-focused attitude and an understanding interpersonal tone. Accordingly, the selected items capture teachers’ efforts to acknowledge students’ perspectives while interacting with them in an understanding manner (see Table A1 and Table A2 in Appendix A for the Italian and English versions of the items). The Italian version of the LCQ showed good psychometric properties in previous research at the individual student level (Alivernini et al., 2019; Cavicchiolo et al., 2026).

Students’ Math Achievement was assessed using the scores from the INVALSI national standardized tests of mathematics developed using Item Response Theory (IRT) models (INVALSI, 2015). The test covered several content domains, including algebra, geometry, statistics, and calculation, and consisted primarily of multiple-choice items, with a smaller proportion of open-ended items requiring a single correct response. Each item response is coded dichotomously as correct (1) or incorrect (0). Students’ total scores were subsequently normalized to a 0–100 scale to facilitate interpretation of the results.

Students’ immigrant background was classified according to the OECD framework (OECD, 2014) into three categories. Native students were defined as those born in Italy with at least one parent also born in Italy. First-generation immigrant students were those born abroad to parents who were also born abroad, whereas second-generation immigrant students were those born in Italy to parents born abroad. For the purposes of the present analyses, immigrant background was entered into the DL-ML-SEM as a dichotomous student-level covariate, coded 0 for native students and 1 for students with an immigrant background (i.e., first- and second-generation immigrant students).

Students’ sex was assessed via self-report, using a single item from the national assessment questionnaire (Are you female or male?’), with response options male and female.

School type was classified into three categories according to the Italian upper secondary education system: Liceo, Technical Institute (Istituto Tecnico), and Professional Institute (Istituto Professionale). Licei provide a broad academic education designed primarily to prepare students for higher education. Technical Institutes combine general education with specialized technical and scientific instruction, preparing students for both tertiary education and entry into skilled occupations. Professional Institutes offer a more vocationally oriented curriculum, with a stronger emphasis on laboratory activities, practical learning, and preparation for employment, while also providing access to tertiary education. For the purposes of the present analyses, school type was included as a classroom-level covariate in the DL-ML-SEM as a categorical variable, with Liceo serving as the reference category and Technical Institute and Professional Institute represented by two dummy variables.

### 2.3. Data Analysis

As a preliminary phase of the analysis, descriptive statistics and Intraclass Correlations (ICC) for the study variables and correlations among them were computed. Consistent with the hierarchical structure of both the aims and the data in the present study (i.e., students nested within classrooms), analyses were conducted using doubly latent models integrating multilevel and structural equation modeling approaches (DL-ML-SEM; Marsh et al., 2012; Morin et al., 2014). This approach enables the simultaneous modeling of observed and latent variables while providing separate, theoretically unbiased estimates of effects at each level of analysis. Analyses were performed in Mplus 8 (Muthen & Muthen, 2019) using the Robust Maximum Likelihood (MLR) estimator and specifying two levels: a within-classroom level (L1) and a between-classroom level (L2). Model fit was evaluated according to conventional criteria, using both the MLR chi-square test statistic and standard fit indices, including the Comparative Fit Index (CFI), the Root Mean Square Error of Approximation (RMSEA), Standardized Root Mean Square Residual (SRMR). Missing responses to questionnaire items ranged from 0.9% to 1.5% and were handled using Full Information Maximum Likelihood (FIML) estimation as implemented in Mplus.

In accordance with the aims of the present study, the first stage of analysis involved conducting a Multilevel Confirmatory Factor Analysis (ML-CFA) to examine the multilevel measurement model of autonomy-supportive climate. At both L1 and L2 it was modeled as a latent construct measured by the four items of the LCQ. Students’ responses are partitioned into two distinct components: one representing the shared classroom-level perception of autonomy-supportive climate (i.e., the aggregated L2 variable, autonomy-supportive climate), and the other representing within-class individual differences in students’ perceptions of autonomy support (i.e., the residual L1 variable, autonomy-supportive microclimate). This approach is consistent with the conceptualization of autonomy-supportive climate as a classroom climate construct, whereby students within the same classroom provide ratings of a common Level 2 construct.

For methodological and interpretative purposes, cross-level measurement invariance was subsequently tested by comparing a model in which factor loadings were freely estimated across levels with a model in which factor loadings were constrained to equality across levels (Mehta & Neale, 2005). Measurement invariance was considered supported when the constrained model showed little or no decrement in fit according to the goodness-of-fit indices. Finally, intraclass correlation coefficients (ICC1 and ICC2; Lüdtke et al., 2008) were computed. ICC1 estimates the proportion of variance in teachers’ autonomy support attributable to the group level. ICC2 estimates the reliability of the aggregated student-level scores, thereby providing an indication of the degree of consensus among students within the same class regarding the autonomy support provided by their teachers.

In the second stage of the analysis, DL-ML-SEM (Marsh et al., 2012; Morin et al., 2014) was employed to examine the associations between autonomy-supportive climate and mathematics achievement. Autonomy-supportive climate and mathematics achievement were specified as multilevel constructs with both Level 1 and Level 2 variance components using the latent aggregation procedure implemented in Mplus, which estimates these components as independent of one another (Morin et al., 2014). Consistent with the conceptualization of autonomy-supportive climate as a classroom climate construct, the shared component of students’ ratings was modeled as a latent Level 2 construct. Accordingly, its association with mathematics achievement was estimated at the classroom level without adjusting for the corresponding Level 1 component (Morin et al., 2014). This approach allowed us to estimate both the association between autonomy-supportive classroom climate and the classroom mean in mathematics achievement (i.e., the climate effect) and the association between students’ unique perceptions of autonomy support and their individual mathematics achievement (i.e., the microclimate effect). To control for potential confounding influences, students’ sex and immigrant background were included as student-level covariates, whereas school type was included as a classroom-level covariate.

## 3. Results

Correlations between the study variables, their descriptive statistics and Intraclass Correlations (ICC) are reported in Table 1 (see Table A3 in Appendix A for the Italian and English versions of the items). Between-level correlations among the study variables are presented in Table A3. 

In the first stage of the analysis, the multilevel CFA indicated that the measurement model of autonomy-supportive climate demonstrated a good fit to the data: χ^2^_(4)_ = 94.28, *p* < 0.001, CFI = 0.993, RMSEA = 0.029; SRMR-within = 0.013; SRMR-between = 0.091. Fit indices remained largely satisfactory in the factorial invariance model, with only minor changes, mostly attributable to the SRMR-between: χ^2^_(4)_ = 198,57 *p* < 0.001, CFI = 0.985, RMSEA = 0.032; SRMR-within = 0.017; SRMR-between = 0.129. Comparison of the two models yielded ΔCFI = 0.008 and ΔRMSEA = 0.003, both of which were below the recommended cutoff of 0.010 (Cheung & Rensvold, 2002), supporting the constrained model. Regarding the SRMR, Hsu et al. (2015) cautioned against strictly relying on conventional cutoff values (SRMR ≤ 0.08; Hu & Bentler, 1999) when evaluating multilevel models. Accordingly, we inspected the residuals to investigate the slightly elevated SRMR-between value. This inspection revealed only small residuals distributed across the model, with no evidence of a specific source of model misfit, suggesting that the value of the SRMR-between reflected minor, diffuse discrepancies rather than localized misspecification. Finally, the factor loadings for the invariance model (reported in Table A4 in Appendix A) were all statistically significant (*p* < 0.001) and exceeded 0.45. Taken together, these findings supported retaining the model with invariant factor loadings across levels, despite the statistically significant chi-square test, given the well-documented sensitivity of the chi-square statistics to large sample sizes (Hu & Bentler, 1999). Overall, the results supported the conclusion that the construct of autonomy-supportive climate exhibited the same factor structure at both the between-class and within-class levels.

This model was subsequently used to compute ICC1 and ICC2 (Lüdtke et al., 2008). ICC1 was 0.22, indicating that 22% of the variance in students’ perceptions of autonomy-supportive climate was attributable to between-class differences. ICC2 was 0.83, indicating good reliability of the aggregated student perceptions at the classroom level (Lüdtke et al., 2008). The ICC(2) values for the four items were 0.71, 0.69, 0.60, and 0.71 for Items 1 through 4, respectively.

**Figure 1:**
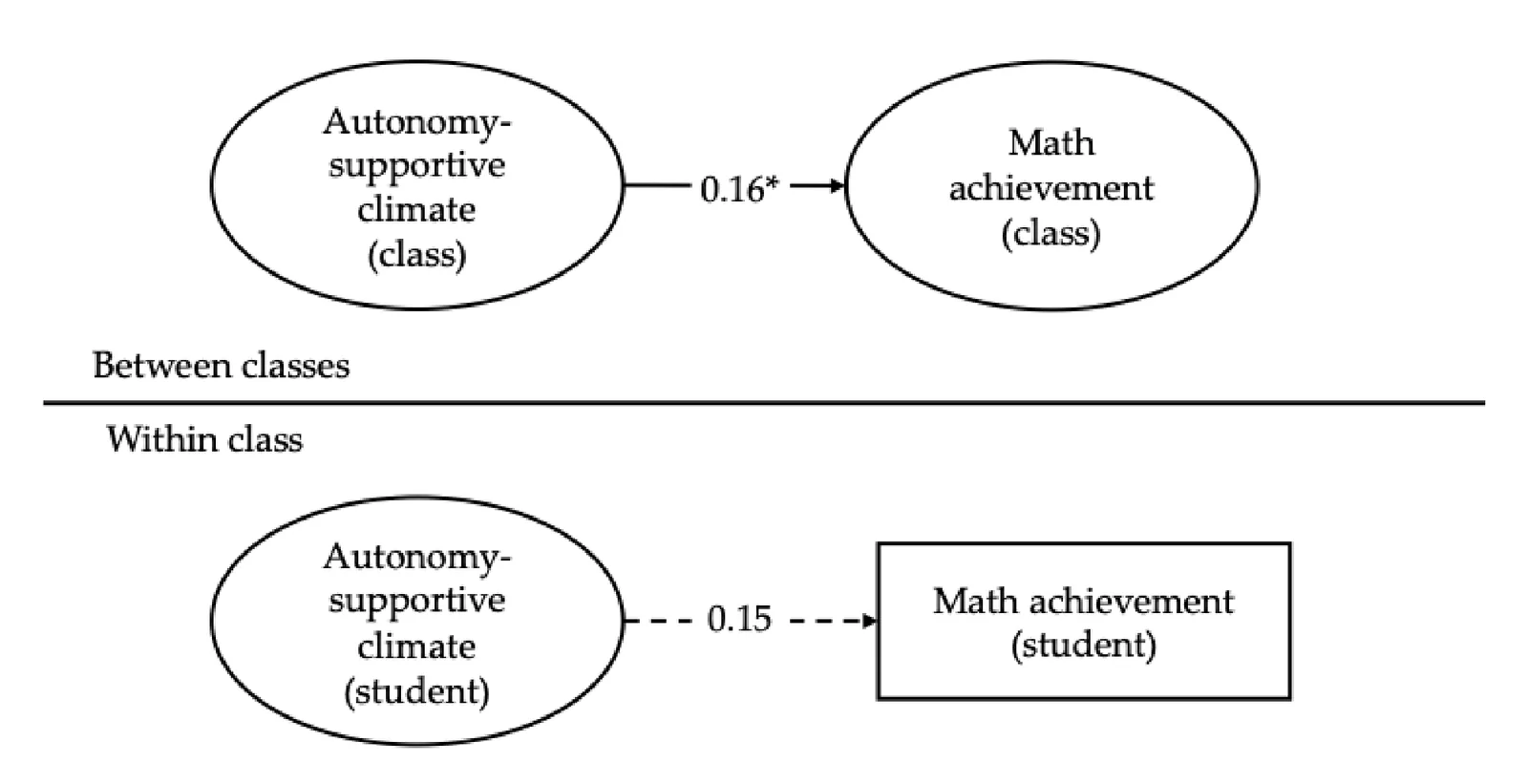
Main results of the DL-ML-SEM. Standardized coefficient. Dashed lines indicate paths that are not statistically significant (* *p* < 0.001).

The main results of the DL-ML-SEM conducted in the second stage of the analysis are presented in Figure 1, whereas the complete results are reported in Table A5 in Appendix A. The tested model demonstrated a good fit to the data: χ^2^_(13)_ = 639.37, *p* < 0.001, CFI = 0.960, RMSEA = 0.031; SRMR-within = 0.017; SRMR-between = 0.131. As in the previous analysis, given the slightly elevated SRMR-between value, we inspected the residuals. This inspection revealed only small residuals distributed across the model, with no evidence of a specific source of model misfit, suggesting that the elevated SRMR-between reflected minor, diffuse discrepancies rather than localized misspecification.

The results of the DL-ML-SEM showed that autonomy-supportive climate was positively and significantly associated with mathematics achievement at the classroom level (β = 0.16, *p* < 0.001) and accounted for a significant proportion of the between-class variance in mathematics achievement (5.3%, *p* < 0.001), after controlling for school type differences (see Table A5 for the complete results). At the within-class level, no statistically significant association emerged between autonomy-supportive climate and mathematics achievement (*p* = 0.064), after controlling for students’ sex and immigrant background differences (see Table A5 for the complete results). The difference between the within- and between-level path coefficients was tested formally and found to be statistically significant (z = 5.42, *p* < 0.001).

## 4. Discussion

Despite extensive evidence demonstrating the importance of autonomy-supportive climate for a wide range of positive student outcomes, as well as its central role in both Self-Determination Theory (Ryan & Deci, 2017) and motivational climate theory (Robinson, 2023), its multilevel structure and its multilevel effects on students’ mathematics achievement remain largely unexplored. The present study addressed this limitation by applying DL-ML-SEM to data from a large representative sample of 10th-grade Italian students

The first aim of the study was to empirically examine the multilevel structure of autonomy-supportive climate while estimating both within- and between-classroom variability. The ML-CFA results provided support for the multilevel measurement model of autonomy-supportive climate, thereby offering evidence for the factorial validity of the construct within a multilevel framework. Furthermore, the establishment of cross-level measurement invariance suggested that the construct had equivalent meaning at the individual and classroom levels. In addition, the ICC1 results indicated that a meaningful proportion of the variance in students’ perceptions of autonomy-supportive climate (approximately 22%) was attributable to differences between classrooms. This finding supported the interpretation of autonomy-supportive climate not only as an individual perception, but also as a shared classroom-level characteristic. Accordingly, the results provided strong empirical evidence for examining the influences of autonomy-supportive climate both at the individual student level (i.e., microclimate effects) and at the classroom level (i.e., climate effects). Moreover, the ICC2 value of 0.83 indicated good reliability of the aggregated classroom-level scores, further supporting the reliability of this measure for assessing classroom autonomy-supportive climate. This result also suggested substantial agreement among students regarding their teachers’ autonomy-supportive behaviors. Such findings support the conceptualization of autonomy-supportive climate as a shared classroom experience that contributes to shaping the motivational climate of the classroom (Robinson, 2023). Furthermore, these findings are consistent with observational research showing that teachers typically spend most classroom time providing whole-class instruction rather than interacting individually with students (OECD, 2020), and may also reflect a well-functioning classroom social environment. Indeed, previous research has suggested that higher levels of consensus in shared group perceptions are associated with more cohesive and adaptive social environments, whereas low consensus may generate within-group stress and strain interpersonal relationships (Bardach et al., 2019).

The second aim of the present study was to examine the associations between autonomy-supportive climate and students’ mathematics achievement at both the individual and classroom levels. The well-fitting DL-ML-SEM indicated that autonomy-supportive climate was positively and significantly associated with mathematics achievement only at the classroom level. This classroom-level association suggests that, after controlling for school type, classrooms characterized by higher levels of autonomy-supportive climate also exhibited higher levels of mathematics achievement. The magnitude of this association is consistent with that reported in previous multilevel studies examining the associations between teachers’ classroom practices (e.g., individual learning support and cognitive activation) and students’ performance on standardized mathematics achievement tests after controlling for school type (e.g., Baumert et al., 2010; Kunter et al., 2013). In contrast, individual students’ perceptions that deviated from the shared classroom climate were not associated with their own mathematics achievement after controlling for students’ sex and immigrant background. Thus, although the findings revealed substantial within-class variability in students’ perceptions, they did not provide evidence for a microclimate effect on mathematics achievement.

In addition to being consistent with previous studies reporting a positive association between autonomy-supportive climate and mathematics achievement (Fu et al., 2023; Wei et al., 2020; Wei & Zhang, 2025), the present findings extend the literature by clarifying the multilevel nature of this association and by examining separately the associations of classroom climate and individual microclimates with mathematics achievement. The classroom-level association observed here reflects the shared, consensually perceived climate of the classroom as an emergent group-level construct, rather than the appraisal of a single mathematics teacher’s behavior. To the best of our knowledge, this is the first study to integrate a DL_ML_SEM analytical approach with the motivational climate theory framework to examine the association between autonomy-supportive climate and mathematics achievement. Moreover, the classroom-level association, together with the absence of evidence for a microclimate effect, emerged in a large, nationally representative sample after controlling for students’ characteristics, including sex and immigrant background, as well as school type. However, given that research on the relative contributions of classroom climates and microclimates to educational outcomes is still developing (Johnson et al., 2024), and that the associations between instructional quality and students outcomes may varies across countries, age groups, subjects and research designs (Senden et al., 2021), further studies are needed to determine whether this pattern of findings can be replicated and to better understand the role of autonomy-supportive climate in students’ mathematics achievement.

Despite its strengths, this study has some limitations that should be acknowledged. First, although the study was based on a comprehensive dataset including a large representative sample of 10th-grade students, further research is needed to examine whether the present findings generalize across other school grades, cultural contexts, subjects and research designs. Investigating autonomy-supportive climate in different educational systems and cultural settings would help determine the extent to which the patterns observed in the Italian sample are generalizable to students from other countries. This is particularly relevant with respect to the role of classroom microclimate: although the absence of a significant association was observed in the present study, further research is needed to determine whether this pattern is stable across different samples and contexts. Second, although the hypothesized direction of the associations between variables—namely, that autonomy-supportive climate promotes students’ academic achievement—is grounded in well-established theoretical frameworks and supported by a substantial body of empirical research, the cross-sectional nature of our data limits the possibility of drawing causal inferences. Longitudinal studies are still needed to clarify the directionality and potential reciprocal nature of these relationships. Finally, although the four-item measure of classroom autonomy-supportive climate demonstrated a clear multilevel structure and adequate reliability in the present study, its brevity inevitably limits the breadth of the construct that can be assessed. Specifically, the selected items represent key aspects of autonomy-supportive teaching but may not encompass the full range of autonomy-supportive behaviors identified in more recent conceptualizations of motivational teaching (e.g., Ahmadi et al., 2023; Germani et al., 2026). Future research should further examine the validity of this brief measure and investigate whether broader item sets capturing additional behaviors provide incremental explanatory value.

## 5. Conclusions

By applying DL-ML-SEM to a large representative sample of Italian secondary school students, the present study contributes to the literature on autonomy-supportive climate by providing empirical support for its multilevel structure and by examining the distinct roles of classroom climate and student-level microclimate in relation to students’ mathematics achievement. Our findings regarding the measurement properties of autonomy-supportive climate within a multilevel framework provide important support for its use in future research on motivational climate processes, as well as in schools seeking to monitor and improve autonomy-supportive teaching practices across classrooms. Moreover, the positive association between autonomy-supportive climate and mathematics achievement at the classroom level, together with a not significant association at the individual level, suggests that the collective motivational environment created by teachers may be more important for academic achievement than students’ individual perceptions of teacher support that diverge from the shared classroom climate. These findings also highlight the importance of adopting a multilevel framework in the study of motivational climate processes, as distinctions between classroom climate and individual microclimates cannot be adequately captured using single-level approaches.

The present findings have important implications for educational practice and teacher training. Previous research has shown that interventions designed to increase teachers’ autonomy-supportive practices are generally effective (see Su & Reeve, 2011, for a meta-analysis). Our findings suggest that such programs may benefit from placing greater emphasis on strategies designed to promote an autonomy-supportive climate at the classroom level. Future experimental studies could focus specifically on this type of intervention and examine its effects within a multilevel framework, using measures of autonomy support explicitly designed to capture classroom climate. In addition, future research should examine the possible mediating roles of academic motivation and satisfaction of basic psychological needs in the association between autonomy-supportive teaching and mathematics achievement. Investigating these processes within a multilevel framework may help clarify their differential contributions to students’ achievement. The present findings may be particularly relevant in light of evidence that an autonomy-supportive classroom climate can buffer the negative educational outcomes often experienced by students from low-income families (Alivernini et al., 2023; Cavicchiolo et al., 2026). Together with this literature, our results suggest that promoting autonomy-supportive teaching may represent a promising strategy for reducing educational inequalities and supporting adolescents who are disadvantaged by their socioeconomic circumstances (e.g., Cavicchiolo et al., 2025a, 2025b; Manganelli et al., 2021, 2025; Raimondi et al., 2025a, 2025b).

## Figures and Tables

**Table 1:** Correlations between the study variables, descriptive statistics and Intra-Class Correlations (ICC).

	Item 1	Item 2	Item 3	Item 4	MA
Item 1	—				
Item 2	0.43 ***	—			
Item 3	0.26 ***	0.33 ***	—		
Item 4	0.33 ***	0.44 ***	0.32 ***	—	
MA	0.12 ***	0.08 ***	−0.00	0.06 ***	—
Mean	2.62	2.86	2.73	2.51	46.42
SD	0.85	0.80	0.92	0.83	22.41
ICC	0.12	0.11	0.06	0.12	0.51

Item 1 = In my class, teachers encourage students to ask questions; Item 2 = In my class, teachers listen to how students would like to do things; Item 3 = In my class, teachers provide students choices and options; Item 4 = In my class, students feel understood by teachers; MA = Math Achievement; SD = Standard Deviation; ICC = Intra-Class Correlations. *** *p* < 0.001.

## Data Availability

The data analyzed in the current study are available upon request at: https://serviziostatistico.invalsi.it/en/ (accessed on 10 January 2025)

## Appendix A

**Table A1:** English version of the Learning Climate Questionnaire items used in the present study.

**Think about what happens in your classroom. How much do you agree with the following statements?**		**Strongly disagree**	**Disagree**	**Agree**	**Strongly agree**
Put a cross in only one box for each question.					
Item 1	In my class, teachers encourage students to ask questions	□_1_	□_2_	□_3_	□_4_
Item 2	In my class, teachers listen to how students would like to do things	□_1_	□_2_	□_3_	□_4_
Item 3	In my class, teachers provide students choices and options	□_1_	□_2_	□_3_	□_4_
Item 4	In my class, students feel understood by teachers	□_1_	□_2_	□_3_	□_4_

**Table A2:** Italian version of the Learning Climate Questionnaire items used in the present study.

**Pensa a quello che succede nella tua classe, quanto sei d’accordo con le seguenti affermazioni?**		**Per niente d’accordo**	**Poco d’accordo**	**Abbastanza d’accordo**	**Molto d’accordo**
Barra una sola casella per ogni riga.					
Item 1	Nella mia classe, gli insegnanti incoraggiano gli studenti a fare domande	□_1_	□_2_	□_3_	□_4_
Item 2	Nella mia classe, gli insegnanti ascoltano come gli studenti vorrebbero svolgere le attività.	□_1_	□_2_	□_3_	□_4_
Item 3	Nella mia classe, gli insegnanti offrono agli studenti possibilità di scelta	□_1_	□_2_	□_3_	□_4_
Item 4	Nella mia classe, gli studenti si sentono capiti dagli insegnanti	□_1_	□_2_	□_3_	□_4_

**Table A3:** Between-level correlations among the study variables.

	Item 1	Item 2	Item 3	Item 4	MA
Item 1	—				
Item 2	0.84	—			
Item 3	0.56	0.66	—		
Item 4	0.78	0.89	0.71	—	
MA	0.51	0.35	0.11	0.24	—

Item 1 = In my class, teachers encourage students to ask questions; Item 2 = In my class, teachers listen to how students would like to do things; Item 3 = In my class, teachers provide students choices and options; Item 4 = In my class, students feel understood by teachers; MA = Math Achievement.

**Table A4:** Results of the Multilevel Confirmatory Factor Analysis.

	Factor Loading—Within	SE	Factor Loading—Between	SE
Item 1	0.533	0.007	0.809	0.016
Item 2	0.680	0.007	0.991	0.010
Item3	0.455	0.007	0.800	0.017
Item 4	0.568	0.007	0.872	0.015

Item 1 = In my class, teachers encourage students to ask questions; Item 2 = In my class, teachers listen to how students would like to do things; Item 3 = In my class, teachers provide students choices and options; Item 4 = In my class, students feel understood by teachers; SE = Stander Error. All the factor loadings estimates are standardized and statistically significant (*p* < 0.001).

**Table A5:** Complete results of the DL-ML-SEM at the within level.

**Within class level**				
**Path**	**β**	**SE**	**95%CI**	***p* value**
AU => MA	0.15	0.008	−0.001/0.042	0.064
SEX => AU	−0.04	0.009	−0.062/−0.025	0.000
IMM => AU	−0.02	0.009	−0.036/−0.002	0.027
SEX => MA	0.21	0.008	0.273/0.318	0.000
IMM => MA	−0.08	0.006	−0.131/−0.097	0.000
**Between class level**				
**Path**	**β**	**SE**	**95%CI**	***p* value**
AU => MA	0.16	0.028	0.11/0.22	0.000
TEC => AU	−0.38	0.029	−0.44/−0.32	0.000
PRO => AU	−0.39	0.032	−0.45/−0.33	0.000
TEC => MA	−0.21	0.026	−0.26/−0.16	0.000
PRO => MA	−0.59	0.022	−0.64/−0.55	0.000

AU = Autonomy-supportive climate; MA = Math achievement; SEX = Student’s sex = male; IMM = immigrant background; TEC = Technical institute; PRO = Professional institute; β = standardized coefficient; SE = Stander Error; CI = Confidence Interval.
